# Expectations, concerns and experiences of rehabilitation patients during the COVID-19 pandemic in Germany: a qualitative analysis of online forum posts

**DOI:** 10.1186/s12913-021-07354-8

**Published:** 2021-12-16

**Authors:** Kübra Altinok, Fabian Erdsiek, Yüce Yilmaz-Aslan, Patrick Brzoska

**Affiliations:** 1 Witten/Herdecke University, Faculty of Health, School of Medicine, Health Services Research Unit, Witten, Germany; 2 Bielefeld University, Faculty of Health Sciences, AG3 Epidemiology and International Public Health, Bielefeld, Germany; 3 Bielefeld University, Faculty of Health Sciences, AG6 Health Services Research and Nursing Science, Bielefeld, Germany

**Keywords:** COVID-19, Rehabilitation, Patient-centered care, Health service delivery

## Abstract

**Background:**

The COVID-19 pandemic, as well as efforts to prevent its spread, have had a strong impact on the delivery of rehabilitative services in Germany. While several studies have addressed the impact of these developments on health service providers and COVID-19 patients, little is known about its impact on patients in need of rehabilitative treatment because of other conditions. This study aims to identify expectations, concerns and experiences of rehabilitation patients related to service delivery in this situation.

**Methods:**

Using a qualitative study design, user posts from six German online forums between March and Mid-November 2020 were systematically searched with respect to experiences, concerns and expectations of health care users toward receiving rehabilitative treatment. We used qualitative content analysis with inductive coding as our methodological approach.

**Results:**

Users fearing physical or psychological impairment were concerned about not receiving timely or effective treatment due to closed hospitals, reduced treatments and limited admissions. In contrast, patients more concerned about getting infected with COVID-19 worried about the effectiveness of protective measures and being denied postponement of treatment by the funding bodies. During their stay, some patients reported feeling isolated due to contact restrictions and did not feel their treatment was effective, while others reported being satisfied and praised hospitals for their efforts to ensure the safety of the patients. Many patients reported communication problems before and during their treatment, including concerns about the safety and effectiveness of their treatment, as well as financial concerns and worries about future treatments. Several users felt that their concerns were disregarded by the hospitals and the funding bodies, leaving them feeling distressed, insecure and dissatisfied.

**Conclusions:**

While some users report only minor concerns related to the pandemic and its impact on rehabilitation, others report strong concerns relating not only to their own health and safety, but also to financial aspects and their ability to work. Many users feel ignored and disregarded, showing a strong need for more coordinated strategies and improved communication specifically with funding bodies like health insurance companies and the German pension funds.

## Background

In addition to the COVID-19 pandemic itself, efforts to reduce the number of infections and to protect vulnerable populations have strongly impacted processes and structures in health care services in several ways. Following a rapid increase in infection rates, rehabilitation hospitals had to develop new strategies to deliver safe and effective services and to protect patients and health care workers. In response to this situation, the Deutsche Rentenversicherung Bund (DRV), i.e., the German Pension Insurance Association, which is the responsible funding organization for the majority of rehabilitative treatments provided in Germany, announced a reduction in operation of almost all hospitals nationwide in early March 2020 [1]. Social distancing measures and quarantines were used as primary measures to mitigate infection risk, especially for highly vulnerable population groups, such as the elderly and patients with comorbidities, resulting in the postponement of treatments and the interruption of operations in rehabilitation hospitals [2]. To be able to continue treatment, the majority of rehabilitation hospitals have implemented specific regulations and hygiene precautions, such as mandatory viral or antibody tests before entering a facility, reduced numbers of patients, strict hygiene requirements, visiting bans, reduced group sizes and planned meal times [3, 4].

In the wake of the restrictions implemented to reduce or prevent the spread of COVID-19, the disruption of health care services, forced social isolation at home and movement restrictions also increased the need for rehabilitation persistently [5, 6]. Nevertheless, even in emergencies, many patients have refrained from using health services because of a fear of infection [7]. Compared to 2019, the number of applications for medical rehabilitation services decreased from ca. 1.6 million to ca. 1.4 million in 2020, while the number of actual treatments for adults decreased from ca. 1.0 million to ca. 0.8 million [8].

While several studies focus on improving the treatment and rehabilitation of patients recovering from COVID-19 through newly developed procedures and specific regulations [9–12], few studies provide an insight into the perspective of current health care users who approach the health care system because of non-COVID-related conditions. Considering the importance of developing new measures for potential rehabilitation patients in the future, addressing representations of the current patients’ perception becomes a necessity [13]. Therefore, this study aimed to address the impact of the COVID-19 pandemic on rehabilitation care in Germany from a patients’ perspective. We analyzed discussions on internet forums to identify specific expectations, concerns, and experiences of patients undergoing or in need of rehabilitation in different phases of the COVID-19 pandemic. Insights into these domains can contribute to the development of strategies needed to support rehabilitation facilities meeting the challenge that pandemics and other public health crises present.

## Methods

### Study design and data collection

A qualitative design using inductive qualitative content analysis was employed to analyze posts on German online forums. We used a purposive sampling approach to identify relevant posts. In this respect, six German online forums (names withheld to ensure anonymity of participants) with different thematic foci were chosen for data collection to explore different perspectives of rehabilitation patients. Four of these forums mainly addressed patients with chronic conditions (cancer, psoriasis, depression, stroke), one was a general health and lifestyle forum, and one forum focused on rehabilitation, retirement, and pension planning. Apart from their thematic focus, forums were chosen according to the numbers of active users, numbers of threads and posts, and recent activity (posts in relevant forum sections in the last 48 h before data extraction). Potentially relevant information in these forums was identified through keyword searches using the terms “reha”, “SARS-CoV-2”, “Covid”, “Corona”, “pandemi*” or “lockdown”, covering all postings from the beginning of March to mid-November 2020. The applied time frame was roughly chosen to include posts from the beginning of the pandemic in Germany up to the day of data extraction.

Relevant content was extracted to a text document for data analysis until information saturation was reached and the collection of further user contributions did not provide any new findings. All German threads and comments were translated into English in the coding process, while usernames and nicknames obtained during the data collection were anonymized with a standardized signifier. This approach to create anonymity for the forum users is in line with other studies [14, 15]; therefore, no further ethical evaluation of our study was necessary.

### Data analysis

All posts and comments related to rehabilitation and SARS-CoV-2/COVID-19 discussed in the threads were imported to the qualitative data analysis software MAXQDA to organize and code the data. Qualitative content analysis was chosen for the analysis to systematically classify information and identify themes or patterns from text data [16]. To answer the research question by identifying and categorizing multiple meanings in the text, inductive methods were used during the coding process [17]. Data collection and analysis were carried out simultaneously while the first codes and categories emerged through constant comparison. Two researchers, the author (KA), who is a researcher in public health with experience in qualitative research, and the author (YYA), who is an interim professor (Dr. PH) with several years of experience in qualitative research, jointly conducted open coding, looked for commonalities across the extracted data and created final categories by following an iterative process until theoretical saturation was reached. The coders discussed the evolving list of codes, and in case of disagreement, the discrepancies were regularly discussed with the other team members (FE and PB) until consensus was reached. The final categories were also discussed within the team until a consensus was reached.

### Reflexivity and rigor

Through investigator triangulation and purposive sampling, credibility was ensured [18]. The recruitment process, including website links, thread titles, data production and extraction dates as well as coding reports and memos along with documentation detailing the research aims, design, sampling processes and practices were retained to provide an audit trail. Member checking was not completed since the adopted method and data collection strategies do not require an interaction with the participants; however, negative case analysis was conducted to ensure the validity of the categories for all instances in the data [19].

## Results

Overall, 99 threads and approximately 450 posts were obtained. In our analysis, we identified 9 codes and subcodes from the data, representing five general topics or themes.

### Health concerns due to canceled, interrupted or curtailed treatment

To prevent infections and stop the spread of COVID-19, many rehabilitation hospitals were closed temporarily. The cancellation of admissions and treatments led to anger and concern among health care users. Many health care users indicated anxiety and frustration after incomplete or delayed treatments.

It must have escaped the employees that we are currently in a Corona pandemic. I am really outraged by this behavior. (User #31)

After my hip replacement surgery, I was only able to spend 11 out of 20 days (15 + 5 extension days) in outpatient rehabilitation, because the facility was closed due to Corona. (User #4)

In addition, other patients reported difficulties in getting an appointment due to increased waiting times, travel restrictions, limited admissions to hospitals, and the reduced number of opened hospitals. While patients were living in ongoing pain and with limited mobility after surgery and other treatments, they were unable to or advised not to participate in rehabilitation programs.

I am not angry but rather sad that my treatment in a rehabilitation facility is interrupted. I‘m having a really hard time with my swollen and sore joints. Of course, the newly operated people should get treatment. They can’t go to rehabilitation and there is no follow-up treatment. (User #25)

### Impact of protective measures on treatment quality and effectiveness

In reaction to the persistent threat of the COVID-19 pandemic, several measures were taken to protect patients and staff and to enable hospitals to continue treatment, such as measurements of body temperature, entrance questionnaires about COVID-19 symptoms, visiting bans, a reduction of group therapies, and admission of fewer rehabilitation patients to the hospitals. While some patients starting their rehabilitation found these new regulations acceptable and efficient, others were more skeptical about the necessity and impact of these restrictions. Some current inpatients shared their experience through online forums, detailing their satisfaction with implemented changes and providing advice to other health care users regarding their experiences in the hospitals. These reports mostly focus on which measures were taken and their impact or the lack thereof on the quality of the treatment.

Despite the restrictions, there is a variety of therapies [available], and the hospital organized itself quite well. [ … ] Some leisure activities are not available, but that doesn’t particularly bother me now. The only criticism is the lack of variety in the food. (User #49)

I can only recommend the hospital. At the moment, all employees are working so hard and everything is being done to prevent Corona. The group sizes have been reduced. There are two meal schedules, so that there aren’t too many people in the dining room at the same time. [ … ] The therapists working here are great. (User #50)

In contrast, other users reported dissatisfaction with their stay because of too many restrictions and stated their fears of insufficient or ineffective treatments due to these restrictions. These patients perceived the regulations as potentially hampering their recovery. In consequence, many of these health care users either tried to extend their rehabilitation treatment or to delay it to a later date, hoping for a cessation of the restrictions. The measures were perceived as stressful for the patients. One health care user expressed the excessiveness of the measures while stressing the necessity of relaxation during the rehabilitation program.

My mother is currently in [the hospital] for a convalescence treatment. It has been 4 weeks now, but she does not feel completely fit again. Due to Corona, very few activities were carried out - which prompted her to apply for an extension for medical reasons after consultation with a doctor. How long does it take for such an application to be approved? (User #52)

No meetings, no strolling, and no coffee … I feel very constricted, especially at the Baltic Sea. The rehabilitation should improve psychological well-being, too! (User #51)

Additionally, visiting bans at rehabilitation hospitals were reported as particularly burdensome for some patients, as well as their families and friends. In addition to patients feeling isolated and anxious during their stay, relatives and friends experienced difficulties in receiving information about the health and well-being of the patients directly.

I am just panicking about this hospital and the 5 weeks I'm supposed to spend there in June. Unfortunately, the objection period has expired and I don't know what to do. (User #22)

I am also concerned, because I will miss my husband very much. If you are not allowed to receive visitors because of Corona, this will certainly be very stressful. (User #10)

Due to the Corona crisis I cannot visit him, which makes me so desperate. The only option was to ask the nurse to hold his phone in front of his face and make a video call. (User #11)

### Risk of infection and hygiene concerns

While patients had different opinions on whether the measures taken by the hospitals could have a negative impact on the quality and effectiveness of the treatment, their effectiveness in preventing infections with COVID-19 was doubted by several patients as well. Health care users who had already had prior experience with rehabilitation hospitals shared their observation of the spread of various diseases during their stay. These users were now more concerned about the threat of the COVID-19 pandemic. The fear of COVID-19 led these patients to demand better hygienic measures. In addition, health care users reported a lack of knowledge about the effectiveness of new hygiene regulations in the hospitals, which suggests that they felt unsafe and perceived a lack of relevant information. Several patients also signaled their hesitancy about continuing their treatments in the hospitals.

Can I be sure that there are good hygienic measures in place to prevent the spread of the Corona virus and that sick people are tested? I have been in rehabilitation several times. Sick people come and go all the time. (User #12)

In light of current events (In North Rhine-Westphalia there is a rehabilitation clinic with more than 100 patients and nurses infected with the Corona virus), the question arises, why are these facilities still open at all and why do they continue to accept new patients? (User #13)

### Concerns about the time frame of rehabilitation

Many health care users whose admissions were approved reported trying to postpone or cancel their upcoming stay due to fears of infection and perceived ineffectiveness of the treatment because of the restrictions. Many believed that as a result of the strict regulations, treatment would be inefficient and preferred to delay their appointment to a later date in hopes of a decreased COVID-19 incidence and a subsequent rollback of restrictions. In particular, older adults and patients with chronic illnesses appeared to feel more vulnerable to infections with COVID-19. Although many reported a distinct need for rehabilitation, they perceived delaying their treatment as a more reasonable option instead of risking infection or treatment interruption.

I am supposed to start an inpatient rehabilitation [ … ]. Can the clinic even provide effective therapies, such as kinetotherapeutic baths, relaxation exercises, physical therapies and group therapies? There is a contact ban for public areas. How can that be maintained in this hospital? I am also very afraid of getting infected and of course of loneliness, because visitors are not allowed. So, what can I do to get a postponement for an indefinite period? (User #77)

Due to the new situation caused by the Coronavirus, I am not sure whether I should do it at this time. Gatherings and sports facilities should be avoided. Since all of us in rehabilitation are older and we are already suffering from other illnesses, I think it would be better to postpone my stay. (User #29)

Since I'm really scared about the pandemic, do I have to start a rehabilitation now which takes place in a high-risk area? I have read several times that people were sent home because of Corona. To drop out is not the point and purpose of rehabilitation. At the DRV nobody answers the phone because of Corona. I have already sent an email to move the rehab to a later date. Is it possible that they cancel my rehabilitation?? (User #23)

### Perceived disregard of concerns by multiple stakeholders

Several users reported that their concerns about potential infections, impaired quality of treatment due to restrictive measures, as well as financial concerns, were disregarded by the insurance organization when trying to cancel or postpone treatment. Furthermore, many users reported difficulties in getting relevant information on safety measures, infection risks and conditions of their treatment from rehabilitation hospitals and the insurance organization.

I don't know what to do next. The DRV says there is no problem! My health insurance refuses helping me! I'm just gathering some information here and I know that nobody can help me here. I have already asked for help elsewhere. (User #61)

I withdrew my application for medical rehabilitation due to the Corona pandemic, because I think that the safety precautions won’t be that effective, and rehabilitation would not be as relaxing as it would be under normal circumstances. In my letter, I explicitly pointed out that the pandemic is the reason. Now I have received a letter from the DRV stating that the approval of my treatment will be regarded as irrelevant, since I forfeit the approved treatment. My reasoning was not taken into account at all. (User #65)

After the closure of hospitals and delays of treatments, many patients had to take action to re-arrange the agreement on coverage between insurance companies and rehabilitation hospitals. Since the approval patients received from the insurance organization was valid only for a specific time period, appointment delays led the patients to be concerned about insurance coverage. Similarly, unilateral changes by the insurance organization with respect to the services provided, e.g., offering only outpatient instead of inpatient care, were regarded by patients as health risks, disregarding their fears and safety concerns. These in turn led to additional financial concerns for some patients.

I have received a disability pension due to being fully incapacitated to work for the last 7.5 years. When I applied for the continuation of my disability pension, I was asked to go to rehabilitation and received approval for an all-day outpatient treatment (this was my explicit request). Now the outpatient hospital is refusing due to Corona. Can I be forced to get an inpatient treatment now? My pension has been approved until October and will be discontinued if the treatment is not completed. If I cancel this pension and instead get my old-age pension, will it be as much as what I currently receive? [ … ] Am I obligated to do inpatient rehab even though I explicitly asked for outpatient rehab? What if the disability pension is canceled just because I would not agree to any inpatient rehab? (User #58)

Here, after the new measures in the hospitals were implemented, the requests of health care users for outpatient rehabilitation were denied. The new regulations implemented to address the pandemic also affected the types of care services, the coverage for rehabilitation, and the types of insurance benefits such as disability pension or old-age pension.

## Discussion

Our analysis of posts in six online forums indicates that the COVID-19 pandemic and protective measures implemented to prevent infection create various areas of uncertainty for health care users during the entire process of rehabilitation. Many health care users tried to reduce this uncertainty by sharing their questions, knowledge, and their experiences gained with hospitals and/or the insurance organization online. By evaluating changes in the structure of therapies, rules approved by hospitals, and new regulations concerning hygiene or mealtimes, they provided recommendations and information for other health care users.

Our analysis showed that health care users’ experiences varied greatly concerning several aspects of rehabilitation, but often led to very specific concerns. The cancellation of scheduled treatments and the closure of rehabilitation hospitals in the beginning of the pandemic led to anxiety, sadness, and frustration among several users. Many rehabilitation patients suffered from physical pain, impaired psychological well-being, and limited mobility, which were prolonged as a consequence of the aforementioned measures. Such delays have been shown to adversely affect quality of life, physical functioning and activities of daily living [20]. After the reopening of rehabilitation hospitals, the demand for rehabilitation treatment was further increased by difficulties in getting appointments due to the reduced number of open hospitals, limited admissions and longer waiting times [21].

When hospitals reopened, newly introduced protective measures caused further concerns. The perceived risk of a COVID-19 infection affected patients’ perceptions of protective measures introduced by the hospitals [22]. While some patients considered these measures to be useful and effective, other patients doubted their effectiveness in preventing the spread of COVID-19, leading to further anxiety. Several users also indicated not feeling safe and reported a lack of information. Previous studies indicate that restrictive measures can contribute to subjective uncertainty and are associated with impaired psychological well-being [22–24]. In addition, some patients regarded these measures as potentially hampering their recovery and causing additional psychological distress. For example, visiting bans led patients to feel isolated and lonely due to limitations in social contacts and the substitution of personal meetings with communication via telephone, video telephony or social media. This is in line with other studies suggesting that restrictive measures such as visiting bans or cancelation of group therapies can have detrimental effects on physical and psychological well-being [22, 25]. Accordingly, considering the vulnerability of the rehabilitation population [26], some patients criticize the insufficiency of the restrictions, while some find them excessive.

Health care users in some cases became skeptical of the necessity of many of these measures in the following months, when both the incidence and mortality of COVID-19 decreased. Other patients who had prior experience with rehabilitation treatments still perceived high subjective risk, citing previous infections with other respiratory diseases during rehabilitation as the reason. The perceived threat level of infection determined the perception of protective measures and led to lower acceptance among those feeling less at risk. This is in line with other studies that identified perceived severity of the pandemic as predictors of the perceived importance of hygienic precautions [27].

This led some health care users to try to postpone or cancel their scheduled treatment. Some users reported feeling ignored or disregarded by the insurance organization, which is covering the cost of rehabilitation. In some cases, attempts to switch from inpatient to outpatient treatment or to postpone or cancel treatment were denied or disregarded by the insurance organization, adding additional distress. Users reported being afraid of financial consequences and of being denied treatment at a later point in time, if they chose to cancel their approved rehabilitation. As studies from the USA have shown, the inability to directly access providers for rehabilitation creates delays in treatment and, in our study, in conjunction with uncertain financial consequences of unilaterally canceling scheduled treatment, created additional barriers to utilization of these services [28]. Considering the correlation between insurance coverage and participation in rehabilitation [29], financial concerns are an important factor in underuse of rehabilitation services. In our study, health care users on social media were urgently seeking answers to questions about pensions and coverage of treatment.

These findings suggest distinctive difficulties for health care users in rehabilitation hospitals to obtain reliable, adequate and comprehensive information on central aspects of their rehabilitation in the current situation. The identified areas of uncertainty also indicate the importance of coordinated efforts of health communication to provide clear, unambiguous and consistent information that is easily accessible [30].

### Strengths and limitations

To our knowledge, this is the first study focusing on the experience of patients undergoing or in need of rehabilitative treatment in Germany during the COVID-19 pandemic using online forums as its data source. Online forums and social media can contribute to the empowerment of health care users in obtaining and providing social support, consultation, self-preparation, self-screening, and giving feedback by creating new information [31–33]. In this respect, research using online forums and other types of social media provides the opportunity to observe relationship dynamics between health care users and health providers as well as between different health care users. It can also play a key role in developing effective user-centered strategies and improving user orientation and quality of health services [31, 32, 34]. Online forums and social media can provide rich information [35], and correspondingly, our study reveals disregarded experiences of current health care users and provides an in-depth understanding of patients in need of rehabilitation.

However, using social media as an instrument of analysis has some limitations that need to be mentioned, such as the difficulty of the generalization of the results and unclear characteristics of the population [36]. Six social media forums were used as the data source, which may have limited the variety of perspectives. It is also unclear to what extent the results are applicable to other population groups. Additionally, the utilization of online forums as a data source may have led to a selection bias with respect to the age groups, educational levels, and socio-economic backgrounds included in the study.

## Conclusions

Our study shows that health care users undergoing or waiting for rehabilitation treatment have a multitude of concerns related to their treatment during a pandemic. The risk of infection with COVID-19, prolonged pain, physical and psychological impairment due to delayed or canceled treatments, as well as perceived limitations in the effectiveness of treatment are among the main concerns. Concerns about being infected with COVID-19 during rehabilitation were strongly related to the subjectively perceived threat of the virus and prior experience with rehabilitation treatments, while perceived limitations in the effectiveness of treatments were mostly related to the perception of implemented protective measures. In addition, communication problems and difficulties in the interaction with hospitals and the insurance organization added further concerns about treatment schedules and financial aspects. These findings suggest a strong need for more coordinated efforts and improved communication to mitigate concerns and provide clear information to health care users and patients.

These findings suggest that the use of online forums and other types of social media as an additional tool to gather user feedback, improve communication strategies and provide support and guidance can be beneficial for rehabilitation hospitals, funding providers and other stakeholders. That way, direct access to formal, accurate, and clear information could be accelerated for a diverse range of health care users. Considering not only the communication between health care users and service providers or funding bodies, but also the relations of patients with other health care users, health personnel, and their families or friends, continuity of communication should be supported by digital communication tools. While online forums and social media are well suited for qualitative studies, limitations in generating statistically valid and representative data suggest that their use for quantitative studies is currently questionable. Studies from the USA have shown that urban and more densely populated areas were overrepresented on Twitter [37–39]. Similarly, Pfaffenberger concluded that the reliability and representativity of Twitter data for scientific analyses may be limited [40]. While Eibensteiner et al. conclude that social media polling can help in understanding public health perspectives, they also cite several limitations and challenges to Twitter polls, e.g., restrictions in the number and design of questions and answers, lack of sociodemographic data and a vulnerability to manipulation of votes [41]. Another recent study from Germany found that comparability between a national survey and Twitter data on depressive symptoms during the COVID-19 pandemic was limited and that sampling and access bias in Twitter data could not be ruled out [42]. Further studies are needed to identify best practices for communicating the use and benefits of protective measures and to adapt measures to better address these concerns are warranted.

## Data Availability

The datasets used and/or analysed during the current study are not publicly available due to privacy and data protection concerns (potential de-anonymization) but are available from the corresponding author upon reasonable request.
